# Commentary: “How Much is that Player in the Window? The One with the Early Birthday?” Relative Age Influences the Value of the Best Soccer Players, but Not the Best Businesspeople

**DOI:** 10.3389/fpsyg.2017.00058

**Published:** 2017-01-25

**Authors:** Luca Fumarco, Benjamin G. Gibbs

**Affiliations:** ^1^Research Division RED, National Institute of Statistics and Economic Studies of the Grand Duchy of Luxembourg (STATEC)Luxembourg, Luxembourg; ^2^Department of Sociology, Brigham Young UniversityProvo, UT, USA

**Keywords:** relative age effects, birth date, cut-off dates, school year, bias

Fuelled by Gladwell's (2008), researchers have expanded their gaze beyond sports for evidence of the Relative Age Effect (RAE; Barnsley et al., 1985): that something as arbitrary as the month you were born in has important consequence for later life success. In line with Furley et al. (2016), we agree that any RAE outside of sports deserves closer scrutiny, but unlike Furley et al., we argue that we should not expect to find evidence of RAE for labor market outcomes in the first place, because *there is not sufficient evidence of uniform age cut-offs in school*.

To begin, Furley et al. (2016) investigate the RAE (data from Poli et al., 2015a), and among the 100 richest billionaires (data from Forbes). Loffing (2016) critiques Furley et al. for comparing the soccer players' birthdate distribution to a uniform distribution to find evidence of RAE; this critique should also apply to the analysis on billionaires^1^. In this commentary, we draw attention on one different aspect of Furley et al.'s analysis of billionaires: we believe it to be *a priori* invalid, because it assumes uniformity of school-age cut-off dates that simply does not exist. If we assume that education is the key mechanism of wealth—as does Furley et al.^2^—the correct interpretation of any RAE requires first a clear establishment of the uniform age cut-offs in the given population.

Furley et al. (2016) do not state but implicitly assume that all the businesspeople in their data were educated under the same cut-off date and that this date was January 1st (i.e., the school year equals the calendar year). If such a uniform cut-off existed, it is easy to imagine why it might matter—the maturation gap would mean that the youngest students likely perform worse and/or face lower wellbeing than their older classmates (Bedard and Dhuey, 2006; Schwandt and Wuppermann, 2016) This, it follows, will be mirrored by poor labor market outcomes (Plug, 2001; Black et al., 2011) and wellbeing (Thompson et al., 1999; Matsubayashi and Ueda, 2015). Furley et al. cite no such evidence of RAE for the billionaires.

For a true test of RAE for the billionaires, two conditions should be satisfied. First, individuals in the sample would have to experience the same cut-off dates for school enrollment; if not, for each individual in the data set, the school year has to be adjusted so that the first day corresponds to the cut-off date of the place where the individual was educated (Bedard and Dhuey, 2006). Second, because some billionaires in the Forbes list are foreigners and thus not raised in the US education system, there should be documentation on cut-off dates for their respective country of origin^3^.

While it is generally safe to assume that January 1st is a unique international cut-off date for soccer youth categories (Poli et al., 2015b), this is not true for education. For example, in the US, January 1st is the current cut-off date for only a few states (10 states); moreover, the cut-off date changed several times in some states (6 states), it can change from year to year depending on when lessons start (1 state), and it can change within states as established by local authorities (5 states) (Bedard and Dhuey, 2006). Therefore, people born in the same state, in the same month of the calendar year, but in different years, might belong to different months of the school year^4^. Similar concerns apply to other countries; for information on international cut-off dates refer to Bedard and Dhuey (2006) and the Eurydice website^5^. Therefore, we caution that analyses based on unclear age cut-offs simply do not represent RAE research.

The same attention to cut-off dates should be paid in the sports context as well. Although most countries share the same cut-off date, this was/is not universal. For example, in soccer, Belgium and Germany adopted a January 1st cut-off date in the mid-90's (Helsen et al., 2000; Ashworth and Heyndels, 2007), while Great Britain's cut-off date is still September 1st (Bryson et al., 2014); most countries and US' states apply January 1st as the cut-off date for youth hockey categories, while Minnesota applies August 31st (Fumarco et al., 2016).

We encourage data sharing and the RAE debate more generally. This allows for the RAE scholarship to advance. We hope to add to the debate by reminding scholars of one necessary criteria of RAE research—more detailed information of age cut-offs. In the case of Furley et al., the study should have cited evidence for appropriate cut-off dates before engaging in RAE analyses. Although we agree with Furley et al.'s conclusion that there seems to be no evidence of RAE among billionaires, we offer a different reason why: there is no reason to expect it in the first place.

## Author contributions

LF wrote the first draft of the manuscript, and BG edited and revised it. All authors approved the final, submitted version of the manuscript.

### Conflict of interest statement

The authors declare that the research was conducted in the absence of any commercial or financial relationships that could be construed as a potential conflict of interest.

## Footnotes

^1^See Delorme and Champely (2015) for more details.

^2^Also this assumption is questionable, for two reasons: (i) billionaires might have inherited much of their fortune; (ii) evidence suggests that RAE in education declines over time (Solli, 2012; Larsen and Solli, in press). We are grateful to a reviewer for this insight.

^3^There is also one interpretational problem. The RAE varies with a number of characteristics of the developmental system (in education, Bedard and Dhuey, 2006; Sprietsma, 2010; in sports, Wattie et al., 2015); when we do not account for these characteristics, the interpretation of the RAE is considerably nuanced.

^4^For example, in Arizona 1978, January 1st was the cut-off date; thus, the 1st month of the school year was January, while the last was December. December born pupils who started school in that year were the youngest in their class. In 1979, the state changed cut-off date to December 1st. The 1st month of the school year became December 1979, while the last became November 1980. December born pupils who started school that year were the oldest in their class.

^5^http://eacea.ec.europa.eu/education/eurydice/index_en.php
